# Global health and economic impact of expanding the WHO essential medicines list for psoriasis: a health economic modelling study

**DOI:** 10.1016/j.eclinm.2026.104103

**Published:** 2026-07-27

**Authors:** Fatima Ali, Alison K. Wright, Thomas Graier, Mahira El Sayed, Rachel H. Giles, Jo Lambert, Lars E. French, Henry W. Lim, Darren M. Ashcroft, Peter C.M. van de Kerkhof, Christopher E.M. Griffiths, Sean P. Gavan, Su Mar Lwin

**Affiliations:** a Department of Dermatology, King’s College Hospital NHS Foundation Trust, and King’s College London, London, UK; b Division of Pharmacy and Optometry, School of Health Sciences, Centre for Pharmacoepidemiology and Drug Safety, Faculty of Biology, Medicine and Health, University of Manchester, Manchester, UK; c Department of Dermatology and Venereology, Medical University of Graz, Graz, Austria; d Ain Shams University Cairo, Egypt; e Department of Patient Engagement, Patvocates, Riemerling, Germany; f Department of Dermatology, Ghent University Hospital, Gent, Belgium; g Department of Dermatology and Cutaneous Surgery, University of Miami Miller School of Medicine, Miami, FL, USA; h Department of Dermatology, Henry Ford Health, Detroit, MI, USA; i Division of Pharmacy & Optometry, School of Health Sciences, Faculty of Biology, Medicine and Health, University of Manchester, Manchester, UK; j Department of Dermatology, Radboud University Medical Center, Nijmegen, Netherlands; k Centre for Dermatology Research, NIHR Manchester Biomedical Research Centre, University of Manchester, Manchester, UK; l Division of Population Health, Health Services Research and Primary Care, School of Health Sciences, Manchester Centre for Health Economics, Faculty of Biology, Medicine and Health, The University of Manchester, Manchester, UK; m St John’s Institute of Dermatology, School of Basic and Medical Biosciences, King’s College London, London, UK; n St John’s Institute of Dermatology, Guy’s and St Thomas’ NHS Foundation Trust, London, UK; o International League of Dermatological Societies, London, UK; p International Psoriasis Council, Santa Rosa, USA; q Global Psoriasis Atlas, Manchester, UK

**Keywords:** Psoriasis, Biosimilars, World Health Organization (WHO), Essential medicines list, Health economic modelling, Quality-adjusted life-years

## Abstract

**Background:**

Psoriasis affects at least 60 million people globally, but access to effective biologic treatments is limited, particularly in low- and middle-income countries. This study models the incremental global health and economic impact of large-scale access expansion for adalimumab and ustekinumab biosimilars following their inclusion on the World Health Organization (WHO) Essential Medicines List (EML) for psoriasis, as recommended by the 25th WHO Expert Committee on Selection and Use of Essential Medicines.

**Methods:**

An empirical decision-analytic health economic model was developed to estimate the incremental costs and quality-adjusted life-years (QALYs) over a five-year horizon, using a 3% discount rate. The model compared the hypothetical current status quo with an EML expansion scenario for a target population of 8.9 million adults with moderate-to-severe psoriasis refractory to conventional systemic therapies.

**Findings:**

Expanded biosimilar access under EML implementation was simulated to generate an incremental 0.625 QALYs per patient at an additional cost of $13,973 USD over five years. Extrapolated to the global target population, this policy could deliver a total health gain of 5.56 million QALYs, with an associated incremental budget impact of $124.35 billion USD over five years. One-way and probabilistic sensitivity analyses confirmed the robustness of these findings: probabilistic analysis mean of 5.67 million QALYs (95% Credible Interval [CrI] 0.38–12.84 million). These findings should be interpreted as upper-bound estimates derived from a simplified model that assumes full access in the eligible population, 60% optimal response, sustained five-year treatment benefit with uniform persistence, no switching or waning efficacy, and full biosimilar costs regardless of response.

**Interpretation:**

The recent expansion of the WHO EML for psoriasis to include biosimilars could yield over 5.5 million QALYs globally. The profound health and access implications of the world-wide implementation of this EML expansion will require regional monitoring.

**Funding:**

This study was funded by the International League of Dermatological Societies (ILDS). SML is funded by LifeArc and DEBRA.

**Research in context**

**Evidence before this study**

Psoriasis is a major noncommunicable disease with substantial unmet need, but access to effective biologic therapy remains highly variable across countries, particularly in low- and middle-income countries. Existing literature identifies significant cross-country biologic access variation and includes economic evaluations that are largely concentrated in high-income settings and often based on originator prices. However, no prior global modelling studies have quantified the population-level health and budget impact of expanded access to WHO Essential Medicines listed psoriasis biosimilars, and no studies have systematically estimated the global unmet need across income groups in a single analytic framework.

**Added value of this study**

This is the first decision-analytic health economic modelling study to estimate the potential global health gain and budget impact of the 2025 WHO Essential Medicines List expansion for psoriasis to include adalimumab and ustekinumab. By synthesizing heterogeneous epidemiological estimates, real-world registry inputs, biosimilar pricing evidence, and weighted access estimates stratified by World Bank income classification, the model provides a transparent framework for national procurement and reimbursement planning. Overall, we estimate 5.56 million QALYs gained and US$124 billion budget impact over five years for 8.9 million eligible adults.

**Implications of all the available evidence**

This modelling study converts the WHO EML decision for psoriasis into actionable policy planning strategies and estimates the likely scale of health gain and investment required to practically expand global biosimilar access. Ongoing regional monitoring of real-world uptake, clinical outcomes, affordability, and access impact is essential to ensure appropriate policy translation.

## Introduction

Psoriasis is a globally prevalent chronic immune-mediated disease predominantly affecting the skin associated with a substantial individual and societal burden. Beyond the cutaneous manifestations, psoriasis is a systemic disease associated with a reduced quality of life and important comorbidities including psoriatic arthritis and cardiometabolic syndrome.^1^ Psoriasis reduces quality of life by causing significant physical discomfort (e.g., itch, pain, sleep disturbance), psychological distress (e.g., depression, anxiety, low self-esteem), and limitations in daily functions and social participation (e.g., impact on relationships, work and education).^1^ An estimated 60 million people globally have psoriasis, with increasing prevalence.^2^ There are notable ethnic and geographic variations in psoriasis epidemiology, with prevalence ranging from 0.07% in Southeast Asia to 3.1% in Central Europe. However, 81% of countries lack formal epidemiological data.^3^^,^^4^ There is an essential need for international collaborative temporal psoriasis surveillance using standardized epidemiological research approaches, as exemplified by the Global Psoriasis Atlas (GPA), a joint initiative of the International League of Dermatological Societies (ILDS), the International Federation of Psoriasis Associations (IFPA) and the International Psoriasis Council (IPC).^3^

Biologics have transformed the clinical management and outcomes of moderate-to-severe psoriasis over the last two decades. However, access to biologic agents remains limited in low- and middle-income countries (LMICs).^5^, ^6^, ^7^ In resource-limited countries, conventional systemic therapies (e.g., methotrexate [MTX], ciclosporin), if available, are the mainstay of therapy due to the high cost of originator biologics and inadequate health system infrastructure.^6^ This further widens health and economic disparities since inadequately treated psoriasis, or poor tolerance to systemic treatments, results in significant economic burden on both patients and healthcare systems, with direct and indirect costs varying dramatically.^7^^,^^8^

The increasing global burden of psoriasis combined with substantial global disparities in treatment access presents an essential area for international health policy intervention. The World Health Organization (WHO) Essential Medicines List (EML) provides a global guide for prioritization of medications and procurement decisions to address the most pressing public health needs.^9^ Medications included satisfy selection criteria focused on global disease burden, clinical efficacy, safety and cost-effectiveness. This aims to encourage world-wide accessibility and affordability. Following the ILDS submissions, in 2025 the WHO Expert Committee approved the addition of adalimumab and ustekinumab for psoriasis to the 2025 Model Lists of Essential Medicines, specifying their use for moderate-to-severe disease and recognizing quality-assured biosimilars as therapeutic alternatives.^9^

Biosimilars have an essential role in addressing economic concerns in global access expansion of the WHO EML for psoriasis. Biosimilars have demonstrated comparable therapeutic efficacy and safety to their originator biologic whilst offering substantial treatment cost savings.^8^ Systematic review of randomized-controlled trial data demonstrates no clinically nor statistically significant difference in the efficacy and safety between biosimilars and originators for the treatment of patients with psoriasis.^10^ Cost discounts or rebates ranged from 50 to 80% of the originator’s list price in the case of adalimumab and ustekinumab.^9^ For example, European markets use a competitive tendering system where national health services are able to use their purchasing power to negotiate bulk purchasing deals with biosimilar manufacturers, allowing discounts as high as 60–80% for adalimumab.^9^ In comparison, the US price reductions range from 20 to 30% due to the complex rebate systems and pharmacy benefit managers favoring the originator.^9^

This study aims to estimate, using an empirical decision-analytic health economic model, the incremental global health and economic impact of expanding global access to adalimumab and ustekinumab biosimilars following the recent WHO EML inclusion for psoriasis.

## Methods

### Study design and perspective

An empirical decision-analytic health economic modelling approach is used to provide a formal framework to synthesize evidence on costs and health-related quality of life (QoL) outcomes. The decision context for this analysis is a global policy-planning and budget-impact assessment intended to estimate the scale of potential population-level health gains and financial consequences if WHO EML expansion were translated into broader access for a globally defined target population. By simulating large-scale access to WHO EML-listed biosimilars, we can facilitate informed decision-making at both global and national levels.

The model simulates two identical hypothetical cohorts of adults with moderate-to-severe psoriasis (candidates for systemic therapy). The choice of comparators includes the current status quo compared to global concordance with the recent EML expansion to include adalimumab and ustekinumab biosimilars for psoriasis. The current status quo represents patients who are refractory to conventional systemic therapies (primarily MTX). MTX is assumed to be the predominant systemic agent as it is the most widely used conventional systemic agent. The decision model abstracts this pathway as a single ‘refractory to conventional systemics’ health state rather than modelling each possible alternative conventional drug separately. The analysis adopts a healthcare payer perspective, including direct medical costs of drugs and related healthcare resource utilization, and excludes indirect costs such as productivity losses.

All analyses were performed in R (v4.4.1), and figure generation used dplyr and ggplot2 packages. The static cohort decision model, deterministic one-way sensitivity analyses, and a 10,000 iteration probabilistic sensitivity analysis (Monte Carlo) were implemented with parameter uncertainty modelled using Normal (population counts), Beta (proportions and utilities), and Gamma (costs) distributions, and 95% credible intervals derived from the 2.5th–97.5th percentiles of the simulated output. A goodness-of-fit assessment confirming the internal validity of each distributional parameterization is presented in Supplementary Fig. S1 and Supplementary Table S1.

### Decision-analytic model

**Fig. 1:**
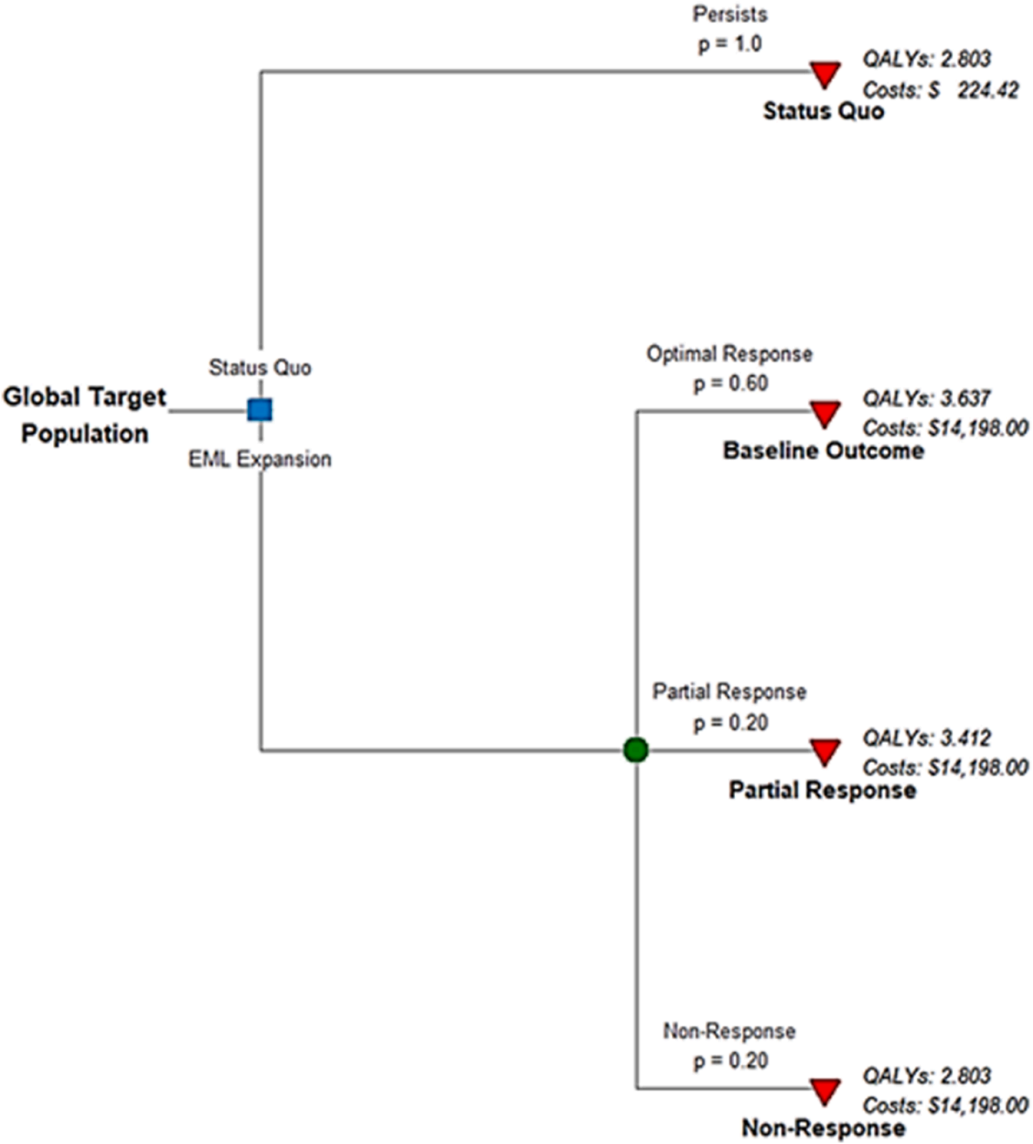
**Decision tree model comparing status quo versus WHO EML expansion response probabilities, discounted per-patient QALYs and costs over 5 years.** Base-case decision tree demonstrating the model structure which compares the status quo and WHO EML expansion for moderate-to-severe psoriasis, where branches display response probabilities and terminal nodes report discounted costs and QALYs over a 5-year horizon from hypothetical treatment initiation. The status quo branch represents persistence to a single terminal value, while the EML expansion branch splits into optimal, partial, and non-response with probabilities p = 0.60, 0.20, and 0.20, respectively. In the model, optimal response corresponds to achieving an absolute PASI of 2 (approximately PASI 90), partial response to an absolute PASI of 5 (approximately PASI 75), and non-response to persistence at an absolute PASI of 15. All costs are in 2024 US Dollars. Abbreviations: EML, Essential Medicines List; QALY, quality-adjusted life-year; p, probability. Blue square = decision node; green circles = chance nodes; red triangles = terminal outcomes.

For a high-level global policy analysis, a static cohort model is used to simulate total costs and quality-adjusted life-years (QALYs) over a fixed 5-year horizon. The model compares two hypothetical arms: the ‘Status Quo’ arm and the ‘EML Expansion’ arm. The Status Quo arm represents the outcome for patients refractory to conventional therapy without access to biologics. It is not an active treatment comparator but rather the baseline against which the EML expansion is measured. As costs of treatments are not dependent on outcome, the model conservatively assumes the full cost of biosimilar therapy is incurred for all patients in the EML expansion arm, irrespective of their response. A decision tree was used for the structure of the decision-analytic model illustrated in Fig. 1.

### Time horizon and discounting

A five-year time horizon is used as this is sufficient to capture the long-term benefits and costs of current systemic/biosimilar therapies for a lifelong condition such as psoriasis. This is also aligned with average biologic drug persistence at 3–5 years.^11^

A 3% annual discount rate was applied to both future costs and QALYs to calculate their present value, consistent with standard health economic procedures.^12^

### Target population and comparators

**Fig. 2:**
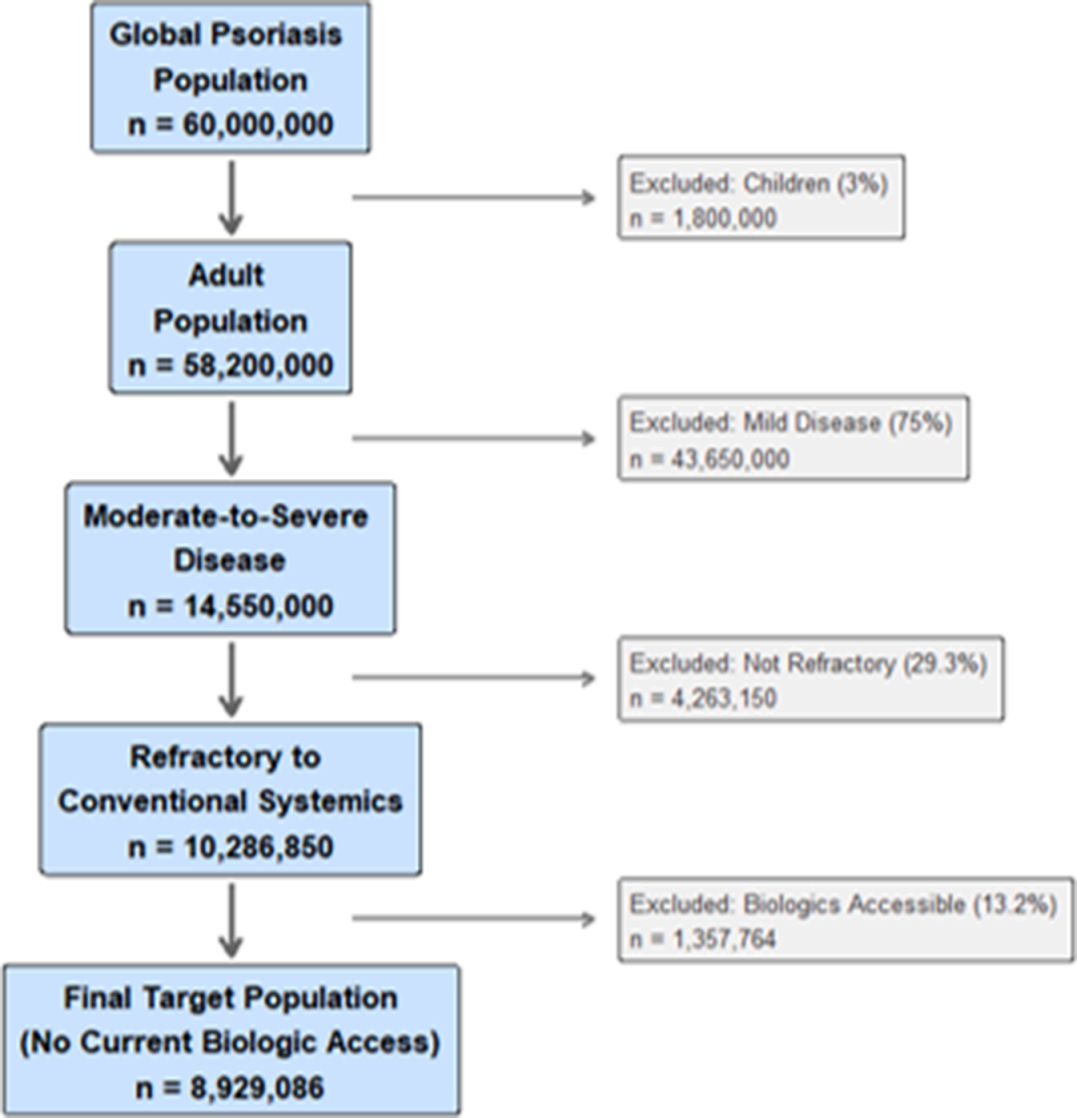
**Derivation of the estimated global target population for WHO EML expansion.** Flowchart of the step-wise derivation of the target patient population for EML expansion. Sequential exclusions are applied to remove subgroups not expected to gain an incremental benefit from the EML expansion. The derivation starts with the global psoriasis population, excludes children, mild disease, those not refractory to conventional systemics, and those with existing biologic access, resulting in the final estimated target cohort that would benefit from EML expansion. In this model, mild disease refers to psoriasis not meeting IPC criteria for systemic therapy; moderate-to-severe disease refers to psoriasis meeting IPC systemic-therapy criteria (i.e., body surface area ≥10%, involvement of high-impact sites, or failure of topical therapy); refractory denotes patients with moderate-to-severe psoriasis inadequately controlled on conventional systemic therapies; and ‘conventional systemics’ are defined as standard non-biologic systemic agents such as methotrexate.”

The hypothetical target population for this model is the global adult population living with moderate-to-severe psoriasis who are refractory to conventional systemic treatments and currently have limited or no access to biologics/biosimilars. This target population would benefit from large-scale extension of biosimilar access aligned with the recommendation of the recent WHO EML expansion to include the adalimumab and ustekinumab and their biosimilars. How the size of this target population was derived is outlined below and in Fig. 2.

The GPA estimates a total of at least 60 million people living with psoriasis globally.^3^ Notably, psoriasis prevalence is higher in adults, with onset peaking at 20–30 and 50–60 years of age.^13^ Given regional heterogeneity and uncertainty in global age-specific proportions, a distribution of 97% adults and 3% children was assumed for the total psoriasis population, producing an estimated global adult population of 58.2 million.^14^, ^15^, ^16^, ^17^ This model estimate is consistent with Parisi et al.’s modelling of the 2017 global adult psoriasis prevalence of 55.8 million (95% uncertainty interval 19.1 to 142.0 million).^4^ This heterogeneity was addressed through deterministic and probabilistic sensitivity analyses.

Published epidemiological studies and systematic reviews demonstrate that the proportion of individuals with psoriasis classified as moderate-to-severe varies widely, from as low as 7%^18^ to as high as 47%,^19^ but commonly reported estimates range from 20 to 30%.^8^^,^^20^, ^21^, ^22^, ^23^, ^24^ However, smaller regional analyses and cross-sectional studies from LMICs report a higher proportion of moderate-to-severe psoriasis.^25^, ^26^, ^27^ A conservative estimate of 25% is applied for the target population. The model targets patients for whom systemic therapy is indicated. It is assumed that the population with moderate-to-severe disease in this model meets the formal IPC criteria to initiate systemic therapy (i.e., Body Surface Area involvement >10%, involvement of high-impact sites, such as the face or hands, or failure of topical therapy).^28^

The model further refines this group to those for whom conventional systemic therapies have failed. MTX is the most widely used conventional systemic therapy for psoriasis. It has high discontinuation rates due to ineffectiveness, adverse events or intolerability. The average duration of use of MTX is 10.1 months with drug survival at 29.3% at 5 years of treatment.^29^ Therefore, it can be assumed that 70.7% of patients who were eligible for systemic therapy are refractory to conventional systemics and would benefit from trialing biologics as a second line systemic therapy if available. We assume this holds in a uniform manner globally in the absence of country-specific drug survival curves.

Populations that already have access to biologics will not experience an incremental benefit from the addition of biologics to the WHO EML and thus, are excluded from the simulated target population. Real-world data about global access to biologics are discordant, particularly in LMICs, which hinders an accurate estimation of global use. To account for this, a weighted population-based model was developed to estimate current global access, as detailed in Table 1. The model estimates a global weighted average biologic access rate of 13.2%.

**Table 1:** Weighted population-based model stratifying global psoriasis and estimated biologic access by world bank national income classification.

Income classification	Proportion of global population (%)	Psoriasis prevalence rate (midpoint %, range)	Estimated psoriasis population (n)	Estimated population refractory to conventional systemics (n)	Estimated biologics access rate (%)	Estimated global biologic access (n)
HIC	16%	2.50% (1.5 to >3.5%)	21,917,808	3,757,753	25.0%	939,438
UMIC	34%	1.25% (0.5–2.0%)	23,287,671	3,992,613	10.0%	399,261
LMIC	40%	0.60% (0.2–1.0%)	13,150,685	2,254,652	1.0%	22,547
LIC	10%	0.30% (0.1–0.5%)	1,643,836	281,832	0.1%	282
Global total/weighted average	100%	1.10%	60,000,000	10,286,850	**13.2%**	**1,361,528**

Weighted population-based model applying psoriasis prevalence to income-group population shares to estimate psoriasis cases, with the refractory cohort defined as adults with moderate-to-severe disease not controlled on conventional systemics under base-case assumptions.

HIC, high-income country; UMIC, upper-middle-income country; LMIC, lower-middle-income country; LIC, low-income country; n, number of people.

Bold values indicate the key weighted summary estimates (global biologic access rate, 13.2%, and corresponding population, 1,361,528) used as base-case inputs in the target population derivation.

To inform model inputs on treatment availability, pooled estimates of global biologic access were stratified by World Bank income groupings.^3^^,^^18^^,^^30^ Reported uptake in high-income countries (HICs) such as US, Italy, Sweden, China, South Korea ranged from 6 to 37% across national registries and insurance databases, supporting a consolidated estimate of 25%.^20^^,^^31^, ^32^, ^33^, ^34^, ^35^ Upper-middle income countries demonstrate heterogeneous access, with figures ranging from 0 to 20% in Chile, Brazil and 4.9% in Colombia; multicenter data further highlight that large patient subgroups effectively have zero access and up to 30% of patients prescribed biologics are unable to obtain them.^6^^,^^36^ A conservative estimate of 10% was therefore applied for upper-middle income countries.^37^ In LMICs, biologic use is rare, with approximately 6% reported in the Philippines, often with assistance programs, 1% in Indonesia and Egypt, and negligible use documented in Ethiopia, and Nigeria, supporting an estimate of 1% for LMICs.^38^, ^39^, ^40^, ^41^, ^42^ For low-income countries (LICs), where published evidence is scarce, proxy data from the GPA and WHO reports indicate that biologics are almost universally inaccessible.^3^^,^^7^^,^^43^ Therefore a pragmatic estimate of 0.1% was applied for LICs. These group-level estimates, shown in Table 1, are intended to reflect the prevailing treatment landscape and to capture substantial regional disparities in biologic access.

Based on these parameters, the final hypothetical global target population representing the unmet need is calculated as 8.9 million people (Fig. 2). Notably, this represents the maximum total potential target population that could benefit from the expansion of the EML; under full and immediate uptake of the EML guidance. The model estimates the potential health gain if access barriers were overcome, not the predicted real-world uptake or the isolated causal effect of listing itself.

### Clinical effectiveness

Psoriasis clinical severity is measured by the Psoriasis Area and Severity Index (PASI). PASI is widely regarded as the gold-standard tool for assessing psoriasis severity, with values ranging from 0 (no disease) to 72 (most severe disease).

The modelled hypothetical ‘status quo’ population are adults with persistent moderate-to-severe psoriasis who are refractory to conventional systemic therapy. An estimated absolute PASI 15 is used as the representative status quo PASI in the primary analysis. This estimate is consistent with large registries such as the British Association of Dermatologists Biologics and Immunomodulators Register (BADBIR) moderate-to-severe psoriasis baseline absolute PASI 15.4 ± 8.1.^44^ Similarly, the German psoriasis registry PsoBest demonstrates mean baseline PASI of 16.1 ± 10.8 at biologic initiation, while the Dutch BioCAPTURE registry reports mean baseline PASI of 13.1 ± 7.6.^45^^,^^46^

In comparison, patients treated under the hypothetical WHO EML expansion scenario were assumed to achieve clinical improvement consistent with commencing biologics/biosimilars of adalimumab or ustekinumab. Absolute PASI of 2 was selected for optimal response. This corresponds closely to a PASI 90 response (>90% improvement from baseline) and concordant with a state of ‘clear’ or ‘almost clear’ skin in 90% of cases.^44^

Clinical evidence demonstrates that adalimumab achieves PASI 90 response rates of approximately 45–70% in real-world settings,^23^^,^^44^^,^^47^ while ustekinumab consistently achieves PASI 90 response rates of 50–70% across multiple studies and over 80% maintaining absolute PASI < 3 in long-term follow-up.^23^^,^^48^^,^^49^ Biosimilars demonstrate equivalent efficacy to their originator biologics in randomized controlled trials and real-world cohort studies.^10^

For modelling purposes, a pooled estimate of 60% achieving absolute PASI ≤ 2 was used, reflecting an assumed equal distribution and optimal response rates for adalimumab and ustekinumab under the EML scenario. The remaining 40% were conservatively split to reflect real-world heterogeneity, where 20% were classified as non-responders/early discontinuations (reverting to the status quo of PASI 15), and 20% achieved a partial response consistent with PASI 75, operationalized as an absolute PASI 5 for utility mapping (Table 2). The hypothetical patients achieving an optimal treatment response in the EML expansion arm were assumed to sustain this benefit over five years. This represents an upper-bound scenario consistent with long-term registry data on average drug persistence, as well as a useful simplification for policy modelling at the global level.

**Table 2:** Per-patient EQ-5D-3L utilities mapped from absolute PASI score and weighted utilities for the status quo and EML expansion arms.

Health State	Absolute PASI score	EQ-5D-3L utility	Distribution (%)	Weighted utility
Status quo arm				
Refractory to conventional therapy	15	0.612	100	0.612
EML expansion arm				
Optimal response	2	0.794	60	0.476
Partial response	5	0.745	20	0.149
Non-responders	15	0.612	20	0.122
Weighted average utility		0.748	100	**0.748**

Utilities mapped from absolute PASI using the PsoReg algorithm with the UK EQ-5D-3L tariff; the EML expansion arm assumes a 60/20/20 response distribution (absolute PASI 2/5/15) and the Status Quo arm assumes absolute PASI 15 for 100% of patients. The weighted average utility for EML expansion is 0.748, which is calculated as: (0.60 × 0.794) + (0.20 × 0.612) + (0.20 × 0.745).

EQ-5D-3L, EuroQol 5-Dimension 3-Level; PASI, Psoriasis Area and Severity Index; QALY, quality-adjusted life-year.

Bold value (0.748) indicates the weighted average utility estimate used as the base-case input for QALY calculations.

### Health utility estimates

Health utility values in this model are calculated through a PASI to EQ-5D-3L fixed-effects regression-based mapping algorithm derived from published longitudinal data of the Swedish Psoriasis Registry (PsoReg) (Table 2).^50^^,^^51^ Predicted EQ-5D-3L values were estimated as a function of PASI, age, body mass index, and psoriatic arthritis, with non-PASI model covariates held at their mean values. We applied these published PASI-specific utility estimates to the modelled health states and did not re-estimate the mapping function in the present study.

The EQ-5D-3L instrument is a widely used generic preference-based measure that captures five dimensions of health with scores ranging between 1 (full health) and 0 (dead), with values < 0 being possible. Utilities were valued using the UK EQ-5D-3L tariff.^50^ The EQ-5D-3L scores are then combined with time spent in each health state to calculate QALYs, defined as the product of duration of time and the corresponding EQ-5D-3L utility score. This represents life expectancy adjusted for the quality of life experienced by patients. Notably, these utilities reflect health-related quality of life associated with psoriasis severity, but do not separately incorporate treatment-related adverse-event disutility. Any health state uncertainty is addressed in the sensitivity analysis.

### Cost parameter estimates

All costs were inflated to 2024 values using an assumed annual inflation rate of 3% and converted to US dollars (USD) using World Bank reported average annual exchange rates of 2024.^52^ The analysis considers direct medical costs, including drug acquisition and healthcare resource utilization.

MTX is regarded as the primary conventional systemic option in moderate-to-severe psoriasis and generally is an inexpensive generic medication. MTX dosing was assumed at 15 mg orally once weekly, consistent with common maintenance dosing.^53^ Unit costs for 2.5 mg tablets from WHO/Health Action International procurement data indicate per-tablet prices of $0.02–0.10 USD and the Management Sciences for Health International Medical Products Price Guide note a buyer median price per 2.5 mg tablet is $0.06 USD.^54^^,^^55^ This translate to an annual MTX drug acquisition cost of approximately $19 USD per patient. This value was taken as the global base case, with sensitivity analysis ranges of $10 to 30 USD. In our model, health system delivery and monitoring costs are accounted for separately under healthcare resource utilization, to avoid double-counting.

Comparatively, biologic and biosimilar therapy costs vary. Originator list prices are higher, often exceeding $10,000 per month in the US.^56^^,^^57^ However, the advent of biosimilars has led to dramatic price reductions, with discounts of up to 80% of list price reported in competitive European markets or country-specific discounts.^58^^,^^59^ In emerging markets, discounts of up to 70% have been observed.^60^, ^61^, ^62^ Adalimumab biosimilars were assumed to follow standard plaque psoriasis subcutaneous dosing (80 mg at initiation, 40 mg at week 1 then 40 mg every other week), and ustekinumab biosimilars standard subcutaneous dosing (45 mg or 90 mg at weeks 0 and 4, then every 12 weeks). A 52-week treatment course with an Adalimumab biosimilar in China was reported to cost approximately $2543 USD.^63^ To reflect a mature and competitive biosimilar market, a conservative annual cost of $3000 USD per patient, from the healthcare payer perspective, is used for the model. Given considerable pricing uncertainty, this parameter is varied extensively in the sensitivity analysis.

Regular monitoring blood tests and dermatology clinic visits may be necessary to manage moderate-to-severe psoriasis, which are generally more frequent for more severe disease. Real-world data demonstrate substantial variation in monitoring costs globally. For this model, an illustrative annual cost of $100 USD is added to the EML expansion arm (assuming more frequent specialist monitoring for blood tests and clinic visits) and $30 USD to the status quo arm.^64^, ^65^, ^66^

A summary of all key model input parameters, including their base-case values, uncertainty ranges, and the distributions used for probabilistic sensitivity analysis, is provided in Table 3.

**Table 3:** Key model input parameters, base-case values, uncertainty ranges, and probabilistic distributions.

Parameter	Base-case value	Range for sensitivity analysis	Distribution (probabilistic)	Source
Population				
Global adult psoriasis population	58 mil	40–65 mil	Normal (mean = 58, SD = 3.5)	3
Proportion with Moderate-to-Severe Psoriasis	25%	20–30%	Beta (α = 25, β = 75)	8,20, 21, 22, 23, 24, 25, 26
Target Population	8.9 mil	6.0–16.5 mil	Derived (Table 1)	Derivation: Table 1
Health utilities				
Status Quo (PASI 15)	0.612	0.55–0.70	Beta (α = 61, β = 39)	45, 46, 47,51,52
EML expansion (60% PASI 2, 20% PASI 15, 20% PASI 5)	0.748	0.70–0.85	Beta (α = 75, β = 25)	45, 46, 47,51,52
Annual costs				
MTX	$19	$10–$30	Gamma (shape = 2, scale = 10)	55,56
Biosimilar	$3000	$1500–$10,000	Gamma (shape = 4, scale = 750)	62, 63, 64
MTX HCRU (lab tests and clinic visits)	$30	$20–$50	Gamma (shape = 3, scale = 10)	65,66
Biosimilar HCRU (lab tests and clinic visits)	$100	$70–$150	Gamma (shape = 4, scale = 25)	65,66

Summary of all key input parameters used in the decision-analytic model, including base-case values used for the primary analysis, the range for sensitivity analysis (used in the one-way analysis), and the distribution used for the probabilistic sensitivity analysis (Monte Carlo simulation). The parameters are grouped by Population, Health Utilities, and Annual Costs. Distribution parameterizations follow conventional forms: Normal for counts, Beta for proportions/utilities, and Gamma for costs, with shape/scale values specified. All costs are in 2024 USD.

EML, Essential Medicines List; PASI, Psoriasis Area and Severity Index; HCRU, healthcare resource utilization; MTX, methotrexate, SD, standard deviation.

### Role of the funding source

This study was funded by the International League of Dermatological Societies (ILDS). Su Mar Lwin is funded by LifeArc and DEBRA. The funding sources had no role in the study design, data collection, data analysis, data interpretation, manuscript preparation, or the decision to submit the work for publication.

## Results

Following the sequential application of exclusion criteria as detailed in the methods and Fig. 2, the final global target population eligible for and able to benefit from the WHO EML expansion was estimated to be 8.9 million individuals.

### Base-case analysis: per patient and global impact

Over a five-year time horizon from treatment initiation, with a 3% discount rate, implementation of the WHO EML expansion decision to include adalimumab and ustekinumab biosimilars for moderate-to-severe psoriasis was projected to generate a discounted incremental QALY gain of 0.625 per patient compared to the status quo. This health gain is associated with a discounted incremental per-patient cost of $13,973.58 over 5 years.

When extrapolated to the 8.9 million eligible individuals globally, the total potential health gain is 5.56 million QALYs, with an associated total incremental budget impact of $124.35 billion over five years. The base-case results are summarized in Table 4.

**Table 4:** Base-case results: per patient and global estimated incremental health and economic impact of EML expansion.

Parameter	Per-patient impact	Total global impact
Total QALYs gained	0.625 QALYs	5.56 million QALYs
Total incremental cost	$13,973.58	$124.35 billion

Base-case incremental outcomes over a 5-year horizon (3% discount). Per-patient results report discounted QALYs gained and incremental cost in 2024 USD, with global results scaling these values to the modelled target population of 8.9 million adults.

QALY, quality-adjusted life-year.

These base-case estimate represents the potential health and budget impact. Hence, estimates should be interpreted as the potential upper-bound impact under an idealized implementation scenario. Specifically, the model assumes that the full eligible population gains access to biosimilars, that 60% achieve an optimal response, 20% a partial response, and 20% no response, that treatment benefit and persistence are maintained uniformly over five years, that no treatment switching or waning efficacy occurs, and that the full cost of biosimilar therapy is incurred irrespective of response.

### Sensitivity analyses

To assess the robustness of the base-case results given the uncertainty surrounding key model parameters, both one-way and probabilistic sensitivity analyses were performed.

#### One-way sensitivity analysis

**Fig. 3:**
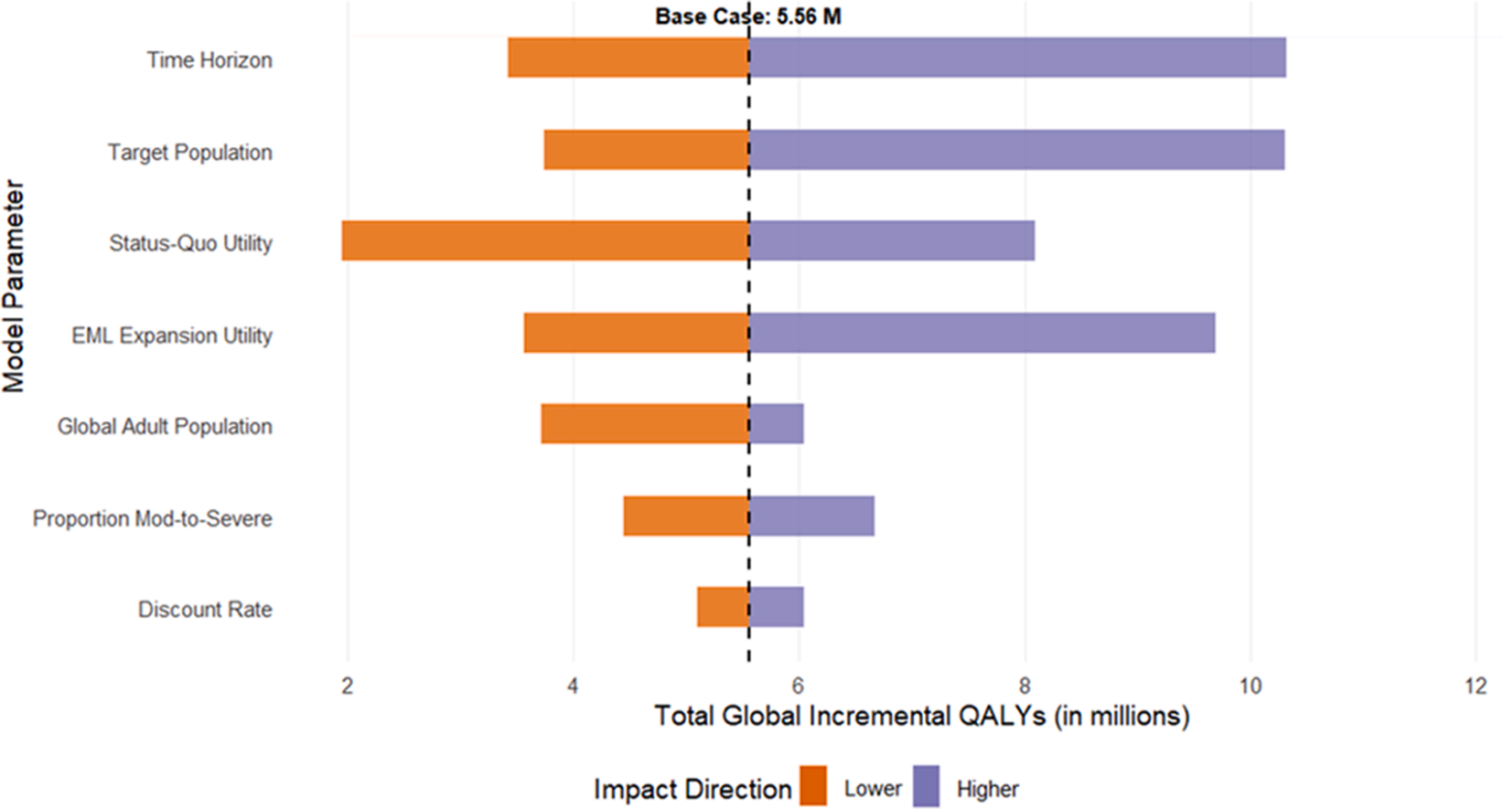
**Tornado diagram one-way sensitivity analysis of total global QALYs gained by varying key model parameters relative to base case.** Effect on total global incremental QALYs when varied across deterministic ranges, where bars to the left indicate lower QALY gains and to the right higher gains relative to the dashed vertical base-case line at 5.56 million QALYs. Colors encode direction of change (orange = lower, purple = higher QALY gained). Abbreviations: QALY, quality-adjusted life-year; EML, Essential Medicines List.

One-way sensitivity analysis of total global QALYs revealed that the model’s outcomes were most sensitive to variations in three key parameters, including identifying variation in time horizon, target population size and utility of achieving PASI responses as the principal drivers for increasing potential incremental QALY gain. The tornado diagram in Fig. 3 visually represents the impact of these and other parameters on the global total QALYs gained. The detailed results of the one-way sensitivity analysis are presented in Table 5.

**Table 5:** One-way sensitivity analysis variables with per-patient and global impact across key input parameters.

Input Parameter	Value	Per-patient incremental cost (USD)	Global incremental cost (USD, billion)	Per-patient incremental QALYs	Global incremental QALYs (million)
Base case	–	**13,973.58**	**124.35**	**0.625**	**5.56**
Global adult population	40 million	NA	83.18	NA	3.72
	65 million	NA	135.17	NA	6.05
Proportion mod-to-severe	20%	NA	99.49	NA	4.45
	30%	NA	149.24	NA	6.67
Target population	6 million	NA	83.84	NA	3.75
	16.5 million	NA	230.54	NA	10.31
Status quo utility	0.55	NA	NA	0.907	8.10
	0.70	NA	NA	0.220	1.96
EML expansion utility	0.70	NA	NA	0.403	3.56
	0.85	NA	NA	1.090	9.70
MTX cost	$10	14,014.80	124.73	NA	NA
	$30	13,923.20	123.92	NA	NA
Biosimilar cost	$1500	7108.58	63.27	NA	NA
	$10,000	46,033.58	409.70	NA	NA
MTX HCRU	$20	14,019.38	124.77	NA	NA
	$50	13,881.98	123.55	NA	NA
Biosimilar HCRU	$70	13,836.18	123.16	NA	NA
	$150	14,202.58	126.40	NA	NA
Discount rate	0%	15,255.00	135.80	0.680	6.05
	6%	12,853.00	114.34	0.573	5.10
Time horizon	3 years	8630.09	76.81	0.386	3.42
	10 years	26,025.65	231.63	1.160	10.32
Realistic Uptake scenarios	25%	NA	31.09	NA	1.39
	50%	NA	62.18	NA	2.78
	75%	NA	93.26	NA	4.17

Deterministic one-way sensitivity for each input parameter at specified values, showing per-patient incremental cost and QALYs, and corresponding global totals scaled to the modelled target population. The base case appears in the first row for reference. Population parameters vary the global adult population, proportion with moderate-to-severe disease, and the derived target population; utility parameters vary status quo utility and EML expansion utility; cost parameters vary methotrexate and biosimilar drug costs and health-care resource use; structural parameters vary the discount rate, time horizon, and illustrative uptake scenarios. Realistic uptake scenarios are also included to scale outcomes to partial adoption of EML-listed biologics, assuming that only 25%, 50%, or 75% of the eligible target population initiates treatment during the time horizon due to phased roll-out and access constraints.

EML, Essential Medicines List; QALY, quality-adjusted life-year; MTX, methotrexate; HCRU, healthcare resource utilization; USD, United States dollars.

Bold values indicate the base-case estimates, used as the reference for the one-way sensitivity analysis.

Specifically, uncertainty in the target population estimate arose from the sequential assumptions used to derive the eligible cohort, including the global adult psoriasis population, the proportion with moderate-to-severe disease, the proportion refractory to conventional systemic therapy, and current biologic access. In one-way sensitivity analysis, varying the global adult psoriasis population from 40 million to 65 million changed projected total global QALYs from 3.72 million to 6.05 million and projected incremental global cost from $83.18 billion to $135.17 billion, whereas varying the proportion with moderate-to-severe psoriasis from 20% to 30% changed projected total global QALYs from 4.45 million to 6.67 million and projected global incremental cost from $99.49 billion to $149.24 billion. When the derived target population was varied from 6.0 million to 16.5 million, projected total global QALYs ranged from 3.75 million to 10.31 million and projected incremental global cost from $83.84 billion to $230.54 billion.

Cost one-way sensitivity analysis showed that the annual biosimilar acquisition cost was the dominant determinant of projected budget impact within the model. At the base-case annual biosimilar cost of $3,000, the model estimated a per-patient incremental cost of $13,973.58 and a total incremental global cost of $124.35 billion over five years. When the annual biosimilar cost was reduced to $1,500, the per-patient incremental cost fell to $7108.58 and the projected global incremental cost fell by nearly 50% to $63.3 billion; when it was increased to $10,000, the per-patient incremental cost rose to $46,033.58 and the projected global incremental cost rose to $409.70 billion. This represents a more than six-fold difference in projected global budget impact across the tested pricing range. By comparison, variation in MTX cost and biosimilar healthcare resource utilisation altered projected global incremental costs by only $0.81 billion and $3.24 billion, respectively, whereas variation in annual biosimilar acquisition cost altered projected global incremental costs by $346.43 billion.

#### Probabilistic sensitivity analysis

**Fig. 4:**
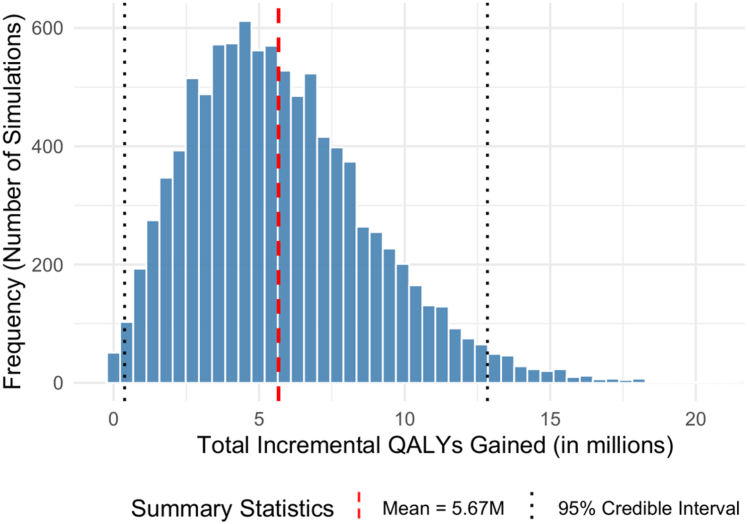
**Probabilistic sensitivity analysis of total global QALYs gained from EML expansion (10,000 Monte Carlo simulations, 5-year horizon, 3% discount).** Monte Carlo simulated distribution of total incremental QALYs in millions; the red dashed line marks the mean (5.67 million) and black dotted lines indicate the 95% credible interval under the probabilistic sensitivity analyses, with all parameters sampled from their specified probability distributions (normal for counts, beta for proportions/utilities, gamma for costs). Abbreviations: QALY, quality-adjusted life-year; EML, Essential Medicines List.

The probabilistic sensitivity analysis, based on 10,000 Monte Carlo simulations, was conducted to simultaneously account for uncertainty across all model parameters (Table 3, and distributional internal validation in Supplementary Fig. S1 and Supplementary Table S1). The results of the probabilistic sensitivity analysis affirmed the robustness of the base-case findings (Table 4). The mean total incremental QALY gain from the simulations was 5.67 million QALYs, with a 95% credible interval ranging from 0.38 million to 12.84 million QALYs. The distribution of simulated outcomes, shown in Fig. 4, indicates a high probability of substantial population health benefit despite the underlying parameter uncertainty.

The wide 95% credible interval (0.38–12.84 million QALYs) arises from propagation of uncertainty in both per-patient utility gains and the size of the eligible global population through the Monte Carlo simulation over a 5-year horizon, causing substantial dispersion when totals are scaled to the population level. The lower tail probabilistic sensitivity analysis simulation near 0.38 million represents the rare worse-case scenario simulations in which the sampled EML utility is very close to the status quo and/or the sampled eligible population is small, producing near-zero total gains. Conversely, the upper-tail simulations near 12.84 million reflect best-case scenarios in which larger per-patient utility gains align with higher eligible population estimates, resulting in maximal total QALY gains.

### Scenario analysis: realistic biosimilar uptake rate

**Fig. 5:**
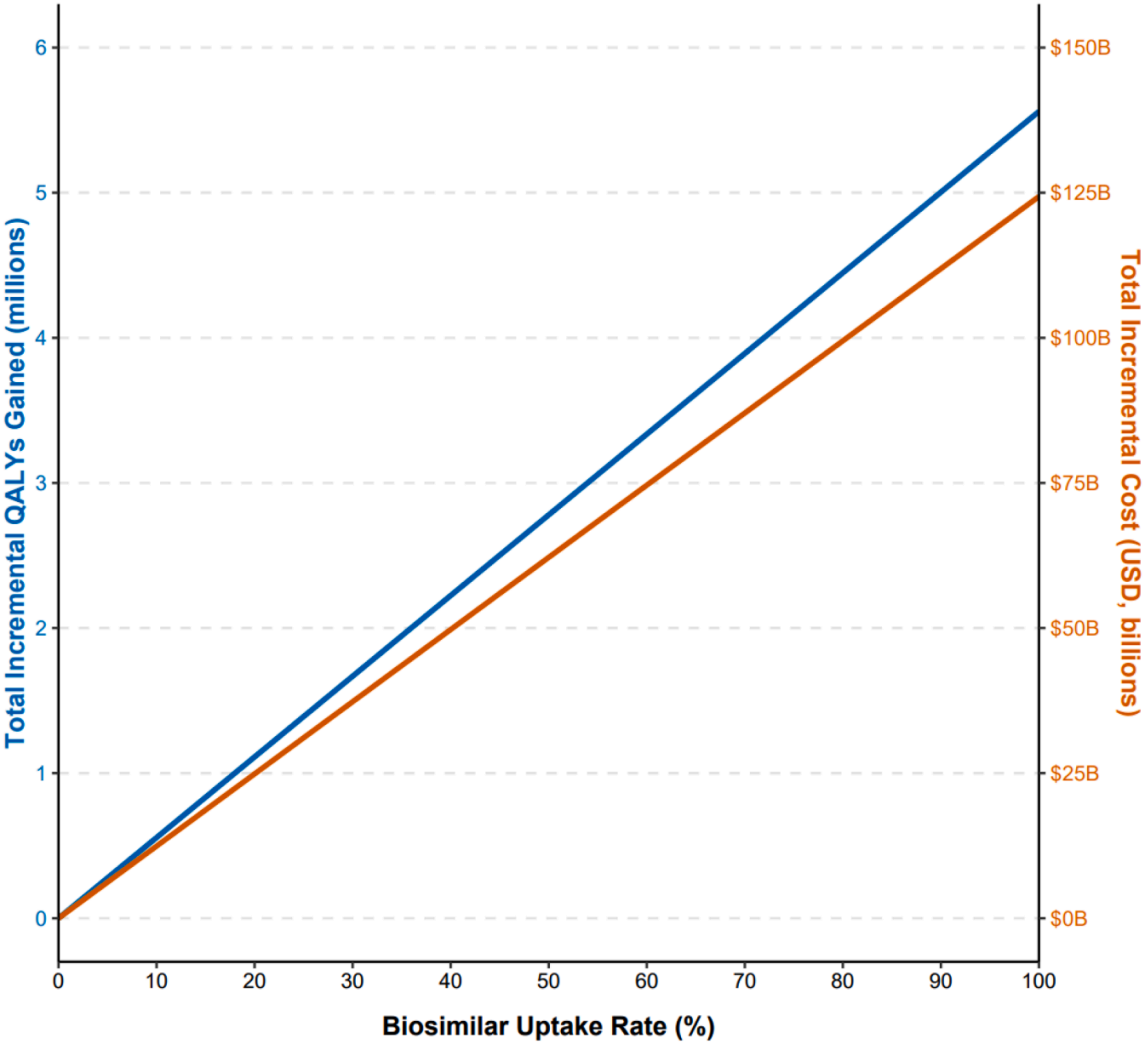
**Global incremental QALY gain and budget impact by biosimilar projected uptake rate for WHO EML expansion.** Projected total incremental QALYs (left y-axis, blue) and total incremental costs in 2024 USD billions (right y-axis, orange) as a linear function of biosimilar uptake among the eligible target population, assuming constant per-patient effects and costs over five years with 3% annual discounting. Axes: x-axis is uptake rate (% of eligible patients initiating EML-listed biosimilars). Abbreviations: EML, Essential Medicines List; QALY, quality-adjusted life-year; USD, United States dollars.

Recognizing that the base-case assumption of 100% uptake of biosimilar use among the eligible population represents an upper-bound ideal situation, a scenario analysis was performed to evaluate the impact of more realistic uptake rates. As highlighted in Fig. 5, the total global QALY gains and incremental costs are linearly proportional to the rate of biosimilar uptake. Under a more conservative assumption of 25% uptake, partial implementation of biosimilar access following EML expansion would still generate a significant health gain of 1.39 million QALYs at a total incremental cost of $31.1 billion over five years. A 50% uptake rate would result in 2.78 million QALYs at a cost of $62.2 billion. This analysis provides a scalable estimate of the potential real-world impact based on implementation effectiveness.

## Discussion

This global health economic modelling study estimates that implementation of the recent WHO EML expansion to include adalimumab and ustekinumab biosimilars could generate a potential health gain of 5.56 million QALYs globally over five years for the 8.9 million adults with moderate-to-severe psoriasis who remain refractory to conventional systemics and currently lack access to these transformative treatments. This significant health benefit is associated with a substantial incremental cost of $124.35 billion, highlighting a critical consideration between health gains and affordability on a global scale. To our knowledge, this is the first study to provide a quantitative global estimate of the health and economic implications of expanding the EML for a chronic non-communicable disease using a formal health economic modelling approach. This provides a data-driven framework for policy decision-making. The model therefore should be interpreted as quantifying the potential health and budget consequences of scaling up access to adalimumab and ustekinumab biosimilars in line with the 2025 WHO EML recommendation, conditional on countries translating this normative signal into financing, procurement, and delivery policies.

At the individual level, the projected QALY gains translate into substantial, clinically meaningful improvements in health-related quality of life, which are aligned with country-specific economic evaluations. The estimated per-patient incremental benefit of 0.625 QALYs over five years (or annual 0.136 QALYs undiscounted) in the model is consistent with published literature on biologics for moderate-to-severe psoriasis. For example, National Institute for Health and Care Excellence (NICE) technology appraisals in England reported one-year incremental QALY gains of approximately 0.143 for adalimumab and 0.156 for ustekinumab. In the present study, we found a similar result, reinforcing the face validity of the per-patient model estimates reported.^67^^,^^68^

While a simulated global mean 5.67 million QALYs (95% Credible Interval [CrI] 0.38 to 12.8 million) gained is significant, it should be interpreted in the broader context of the increasing global psoriasis burden. Studies evaluating the global burden of psoriasis have shown increasing worldwide prevalence and years lived with disability.^69^^,^^70^ However, the aggregate global figures mask important cross-country and income-group differences in access. The model reveals that the vast majority of the 8.9 million people with unmet needs are predominantly concentrated in LMICs. Here healthcare is predominantly financed out-of-pocket (OOP) leading to a large financial burden which can exceed the annual per capita income and cause catastrophic health expenditure where households are forced to forgo basic needs like food and housing to pay for medical care. Catastrophic health expenditure is defined as healthcare costs being >10% of total consumption expenditure.^71^ A 2025 LMIC cross-sectional study of pediatric chronic plaque psoriasis patients found almost 40% of households had catastrophic health expenditure and associated poorer QoL scores.^72^ LMIC data from Colombia also show substantial psoriasis OOP burdens concentrated in medications and transport costs.^73^ In HICs, biologics OOP costs can be significant due to lack of insurance coverage, which can contribute to financial burden and potential non-adherence.^74^ Also, many LMICs face dermatology workforce shortages, limiting access to timely diagnosis and advanced therapies. However, these equity implications are inferred rather than directly measured, as the model does not formally quantify distributional outcomes or financial protection, which would require dedicated distributional or equity-focused modelling approaches.^75^ The profound access and affordability concerns further highlight the importance of expanding the WHO EML.^76^

The one-way sensitivity analysis identified the annual biosimilar cost, target population size, and the magnitude of the utility gain as the most influential parameters, with overall value most contingent on biosimilar price and access. Reducing the assumed annual biosimilar cost to $1500 reduced the global incremental cost by nearly 50% to approximately $63.3 billion, while preserving the full health gain. Whilst the actual biosimilar net prices are confidential, reports of discounts up to 80% off list price provide a viable route to address the global access disparities.^10^^,^^56^^,^^58^ The model’s per-patient and population-level estimates suggest that leveraging biosimilars through inclusion on national and international essential medicines lists could meaningfully reduce the global burden of inadequately treated psoriasis while improving affordability and access. The WHO’s 2025 EML update, which now lists adalimumab and ustekinumab for moderate-to-severe psoriasis, represents an important policy development that may support similar inclusions on national essential medicines lists. This emphasizes that price reductions, driven by robust market competition and strategic procurement, are likely important for EML expansion to be economically viable on a global scale.^61^ Furthermore, a pragmatic affordable step-wise rollout remains impactful, where even a conservative 25% uptake translates to a significant health gain of 1.39 million QALYs at a cost of $31.1 billion, allowing for scalable budget impact planning.^76^ Aligned with global essential-medicines policy, the implementation may need to be paired with integrating these medicines into public benefit packages to curb OOP spending, review of any public displaced funding as a result of EML expansion, meet regulatory requirements for biosimilar approval/procurement, promote biosimilar pricing transparency, and routine reporting against a core set of 24 indicators to track expansion progress and accountability.^76^

From the patient perspective, expanding access to effective biologics has the potential to improve daily life beyond clinical endpoints. For many individuals with moderate-to-severe psoriasis, uncontrolled disease is associated with social stigma, psychological distress, and disruption of work and family roles.^69^ Reliable access to affordable biosimilars could reduce the physical symptoms that drive isolation and depression, restore the ability to participate fully in society, and improve long-term productivity. Patients and advocacy groups consistently highlight that timely access to modern therapies can reduce skin symptoms and psychosocial burden to patients and caregivers.^3^ Access early in the course of psoriasis provides a window of opportunity to reduce the cumulative burden of disease and to prevent cumulative impairment.

This study offers a timely, novel, global perspective with transparent data-driven target-population derivation and comprehensive sensitivity analyses with real-world applicability, which should be interpreted in the context of key methodological assumptions and limitations. Evidence gaps in LMICs required extrapolation of epidemiology, costs, and utilities from high-income country sources, which may miss regional heterogeneity in prices and preferences; however, extensive one-way ranges and a 10,000-iteration probabilistic sensitivity analyses demonstrate that substantial health gains persist across plausible input parameters. The static decision-tree structure does not capture psoriasis or treatment dynamics; and more complex dynamic modelling would require longitudinal data, particularly in LMIC settings.

Model utilities were mapped from PASI using Swedish registry data and valued using the UK EQ-5D-3L tariff and did not include adverse-event disutility, which could shift point estimates, but this was stress-tested in sensitivity analyses varying utility parameters. Also, this approach assumes that PASI-defined severity states are an adequate proxy for utility across settings, which may not fully capture contextual variation in health preferences. The assumptions of sustained biosimilar response over five years, uniform persistence, and neither treatment switching nor waning efficacy simplify the treatment pathway and may overestimate QALY gains. Costs assumed a uniform global biosimilar price and a healthcare payer perspective, excluding indirect costs and spillovers that can represent a large share of psoriasis burden.^77^ The model also conservatively assumes that the full cost of biosimilar therapy was incurred for all patients in the EML expansion arm irrespective of response, which may overestimate incremental drug expenditure where early discontinuation or switching may occur. Hence, the modelled budget impact is conservative relative to a societal perspective but is presented alongside one-way, probabilistic, and uptake-scenario analyses to reflect uncertainty to reflect uncertainty.

Clinically, the base-case estimate of 5.56 million QALYs gained represents a maximum potential, upper-bound scenario. The model assumes a uniform treatment response, full five-year persistence and adherence, and static disease severity for distinct health states. This creates an idealized scenario that represents the maximum potential health gain under optimal conditions, rather than capturing the fluctuations, discontinuations, treatment switching or waning efficacy over time common in clinical practice. Additional health-related QoL gains from concomitant psoriatic arthritis were not modelled, where adalimumab and ustekinumab have demonstrated clinically meaningful lifetime benefits in psoriatic arthritis, with gains of approximately 6.42 and 6.23 QALYs, respectively.^67^^,^^68^

This analysis has several implications for global health policy, including providing quantitative modelling to contextualize the recent addition of adalimumab and ustekinumab biosimilars to the WHO EML. However, the associated cost of implementing this WHO EML expansion highlights a global affordability divide. This encourages policy discussions to extend beyond isolated cost-effectiveness threshold targets, to also consider affordability and financial protection. EML inclusion can be considered a market-shaping intervention which can incentivize biosimilar entry into LMIC markets, create advanced market commitments, facilitate bulk procurement, group price negotiations, generating appropriate infrastructure/technology, and foster sustainable access solutions. The pharmaceutical industry can further support access using tiered pricing, licensing strategies for LMICs and access initiatives.

To enhance the study’s usefulness for national policymakers, the model is designed as an adaptable framework for country-level decision-making. Policymakers in a specific country can utilize local data to generate more relevant local estimates. For instance, they can substitute their own epidemiological data to refine the target population size, use the drug cost sensitivity analysis to interpret results by inputting their locally negotiated biosimilar price, and apply the uptake rate analysis to project their national budget impact based on current and target treatment levels. This adaptability allows our global model to serve as a practical tool for informed national-level procurement, reimbursement, service planning and budget forecasting.

Future research should focus on bridging the evidence gaps highlighted by this analysis. There is an urgent need for LMIC country-specific QoL studies, cost-effectiveness and budget impact analyses. Strengthening psoriasis surveillance in LMICs is essential to reduce uncertainty around disease prevalence, healthcare resource utilization, and treatment outcomes. Implementation also requires a regional monitoring of both outcomes and costs. Country-specific evaluations should also track how WHO EML or national list updates affect medicine prices, availability, and utilization, enabling transparent assessment of impact on access. These analyses should be connected to national guideline updates, ideally in real-time using real-world evidence and cost efficacy. Finally, running policy experiments and research into innovative financing and delivery models is critical to ensure that potential health gains from EML expansion can be realized without imposing undue financial hardship on patients and health systems.

The recent expansion of the WHO EML to include adalimumab and ustekinumab biosimilars for psoriasis could deliver substantial global health gains, with modelling suggesting benefits exceeding 5.5 million QALYs over five years under ideal implementation. However, to appreciate these benefits, countries need fair pricing, efficient procurement, and reliable coverage that can keep biosimilars affordable and help patients stay on therapy, especially in lower-resource settings where out-of-pocket burdens are highest and health system capacity is most constrained. Our findings further support the need for jurisdiction-specific health economic evaluations to inform how biosimilars can be delivered in a cost-effective manner, accounting for local resource availability, budgetary constraints, and health system capacity, particularly within LMICs.

Crucially, the potential impact of expanded biosimilar access following WHO EML listing should also be understood from the patient’s perspective, including embedding of the patient voice in implementation and monitoring to ensure projected health and economic gains translate into meaningful improvements in daily life. This study provides economic support for expansion of national and international EMLs while highlighting that the scale of real-world benefit will depend on biosimilar market sustainability and national policy choices that convert EML into affordable access at scale.

## Contributors

Conceptualization: FA, SML, SPG, CEMG, PCMvdK, AKW, TG, MES, RHG, JL, LF, HWL, DMA.

Methodology: FA, SML, SPG, CEMG, DMA.

Formal analysis: FA.

Software: FA.

Validation: FA, SPG, SML.

Visualization: FA, SPG.

Writing (original draft): FA, SPG, SML.

Writing (review & editing): FA, AKW, TG, MES, RHG, JL, LF, HWL, DMA, PCMvdK, CEMG, SPG, SML.

Supervision: SML, SPG.

Data access and verification: FA and SPG verified the modelling data, tables and visualizations of the manuscript.

All authors read and approved the final version of the manuscript.

## Data sharing statement

The datasets used to develop and validate the model, along with any derived analytical outputs necessary to reproduce the findings, can be accessed upon reasonable request from the corresponding author. Requests will be considered for legitimate research purposes and subject to appropriate data-use agreements.

## Declaration of interests

**FA, AKW, RHG** and **SPG** declare no conflicts of interests. **CEMG** has received honoraria and/or research grants from AbbVie, Almirall, Amgen, Anaptysbio, Artax, Boehringer Ingelheim, Bristol-Myers Squibb, Celltrion, Eli Lilly, GSK, Inmagene, Kyowa Kerin, Johnson & Johnson, Novartis, Nxera Sun Pharma, LEO Foundation, and UCB Pharma. **RHG** received travel grants from Ipsen, Genmab, and previously served as a consultant for Medicom Medical Publishers (2024) who were hired by the ILDS to write two applications for the WHO EML, and received fees from Ipsen, Novartis, J&J, Genmab, Pfizer, MSD, Novo Nordisk, Merck, Incyte, Astra Zeneca, Mirrum, BeOne, BioNTech, and Leo Pharma. **DMA** reports research grants from Boehringer Ingelheim, Bristol Myers Squibb, Janssen, and the LEO Foundation. **TG** has received travel grants from Abbvie, Amgen, Novartis, Johnson & Johnson and Sanofi; speaker honoraria from Amgen, Abbvie, Eli Lilly and Novartis; and consulting honoraria from Almirall. **MES** is an advisory board and speaker for Abbvie, Pfizer, Jansen, Novartis, Eli lilly, Sanofi, EvaPharma, Sandoz, Boehringer Ingelheim. **JL** has received unrestricted grants from AbbVie, Almirall, Celgene, Eli Lilly, J&J, LEO Pharma, Novartis, UCB, and has served as a speaker for AbbVie, Almirall, Bristol-Myers Squibb, J&J, Pfizer, Roche, UCB, and served as a consultant for AbbVie, Almirall, argenx, Bristol-Myers Squibb, Celgene, Celltrion, Eli Lilly, J&J, LEO Pharma, Novartis, UCB. **LEF** has received research funds Leo Pharma, Boehringer-Ingelheim; and consulting fees from Almirall, Janssen, Leo Pharma, Union Therapeutics, Boehringer-Ingelheim, ACImmune, Novartis, Abbvie, UCB, Biotest, AC-Immune, Vaderis Therapeutics, Aldena Therapeutics and Alys Pharmaceuticals. **HWL** has served as an investigator for Incyte, La Roche Posay, Pfizer, and PCORI; as a consultant for ISDIN, Beiersdorf, Ferndale, L’Oréal, Eli Lilly, Zerigo Health, Skinosive, Kenvue, Cantabria Labs, NAOS, and Boehringer Ingelheim; and as a speaker on general educational session for La Roche-Posay, Cantabria labs, Pierre Fabre, NAOS, Uriage, Pfizer, ISDIN, Clinuvel. **PCMvdK** received fees for consultancy service or lecturerships from, Almirall, Eli Lilly, Novartis, Janssen Pharmaceutica, Bristol Mayer Squib, UCB, Boehringer Ingelheim and centrion, Sandoz. **SML** has received honoraria and/or travel and/or research grants from Almirall, Chiesi, Johnson & Johnson, Eli Lilly, L’Oreal, Novartis, Takeda, Sanofi and UCB. SML is also co-founder and trustee of Burma Skincare Initiative Charity.
