# Supplementary material for: Global health and economic impact of expanding the WHO essential medicines list for psoriasis: a health economic modelling study
Source: eClinicalMedicine. 2026 Jul 27;98:104103. doi: 10.1016/j.eclinm.2026.104103 (PMC13445532; doi:10.1016/j.eclinm.2026.104103)
Supplement: Supplementary file 1 [file mmc1.docx]

**Supplementary**

**A.**

**B.**

**C.**

**D.**

**E.**

**F.**

**G.**

**H.**

**Figure S1: Goodness-of-fit assessment for probabilistic sensitivity analysis parameter distributions (n = 1,000 simulations).**
*Panels A to H display histograms of 1,000 values simulated from each assigned distribution: (A) Global Adult Psoriasis Population, (B) Proportion Moderate-to-Severe, (C) Status Quo Utility (PASI 15), (D) EML Expansion Utility (weighted), (E) MTX Annual Cost, (F) Biosimilar Annual Cost, (G) MTX Healthcare Resource Utilisation, and (H) Biosimilar Healthcare Resource Utilization.
The black vertical line denotes the base-case point estimate; dashed grey lines denote the 2.5th and 97.5th percentiles (95% credible interval). Distribution families were assigned by parameter type: Normal distributions for population counts, Beta distributions for proportions and utilities (bounded between 0 and 1), and Gamma distributions for costs (bounded at ≥0). Colour denotes distribution family: blue represents the Normal distribution (population parameters); green represents Beta distributions (proportions and utilities); orange represents Gamma distributions (costs). Simulated means closely reproduce base-case values across all parameters, confirming internal validity of the distributional parameterisation.
Abbreviations: CrI, credible interval; EML, Essential Medicines List; HCRU, healthcare resource utilisation; MTX, methotrexate; PASI, Psoriasis Area and Severity Index; PSA, probabilistic sensitivity analysis; USD, United States dollars.*

**Table S1: Summary of probabilistic sensitivity analysis parameter distributions and internal validity assessment**

| **Parameter** | **Distribution** | **Base-case** | **Simulated Mean** | **95% CrI** |
| --- | --- | --- | --- | --- |
| **Global adult psoriasis population** | Normal | 58mil | 58.1mil | [51.6, 64.7] |
| **Proportion mod-to-severe** | Beta | 25% | 24.8% | [16.5, 33.4] |
| **Status quo utility** | Beta | 0.612 | 0.610 | [0.508, 0.700] |
| **EML expansion utility** | Beta | 0.748 | 0.750 | [0.664, 0.829] |
| **MTX annual cost** | Gamma | $19 | $20.1 | [$3.1, $56.1] |
| **Biosimilar annual cost** | Gamma | $3,000 | $3,024 | [$771, $6,583] |
| **MTX HCRU** | Gamma | $30 | $30.4 | [$7.0, $72.3] |
| **Biosimilar HCRU** | Gamma | $100 | $101.6 | [$30.0, $219.2] |

*For each model input parameter, the assigned distribution family, base-case value, and simulated mean and 95% credible interval derived from 1,000 Monte Carlo draws are presented.
Agreement between the base-case value and the simulated mean, and the plausibility of the 95% credible interval relative to the one-way sensitivity analysis range, confirms the internal validity of the distributional parameterisation.
Abbreviations: CrI, credible interval; EML, Essential Medicines List; HCRU, healthcare resource utilisation; MTX, methotrexate; PASI, Psoriasis Area and Severity Index; PSA, probabilistic sensitivity analysis; USD, United States dollars.*
